# Shared Call Structure and Fine-Scale Spatial Variation in Calls Attributed to *Boesemania microlepis* in the Cambodian Mekong River

**DOI:** 10.3390/ani16213340

**Published:** 2026-10-23

**Authors:** Longfei Huang, Shuqi Long, Qingcheng Tang, Wenyin Wu, Daoping Yu, Yuxi Lian, Minmin Chen

**Affiliations:** 1 Engineering Technology Research Center for Aquatic Organism Conservation and Water Ecosystem Restoration in University of Anhui Province, Anqing Normal University, Anqing 246133, China; 122402@aqnu.edu.cn (L.H.);; 2 Anhui Provincial Key Laboratory of Biodiversity Conservation and Characteristic Resource Utilization in Southwest Anhui, Anqing Normal University, Anqing 246133, China; 3 School of Life Sciences and Food Engineering, Anqing Normal University, Anqing 246133, China

**Keywords:** *Boesemania microlepis*, passive acoustic monitoring, Mekong River, diel calling patterns, call sequence rhythm, spatial variation

## Abstract

Passive acoustic monitoring supports studies of fish activity and habitats in changing tropical rivers, yet cross-site consistency and local variation in calls remain poorly quantified. We monitored four sites (West, Middle, East, and Dock) across approximately 2 km of the Kampi reach of the Cambodian Mekong River. Automated detection and manual review were combined with fish surveys, hydrological observations, and background sound measurements. Published acoustic features and captures at the study sites supported attribution to *Boesemania microlepis*. At all sites, calls shared pulsed, harmonic structure, with site-median harmonic spacing of 114.8–119.1 Hz; 77.3–94.2% occurred at 16:00–20:00 local time. Rates of confirmed received events across daytime and nighttime recordings were 2.32–6.06 calls per minute of valid recording. Date-paired comparisons showed that Dock’s 95% cumulative-energy frequency was 200.0–235.1 Hz lower than at the other sites. Within-sequence call repetition rates at East were 0.327 and 0.241 calls/s lower than at West and Middle, respectively (Holm-adjusted *p* < 0.05). Dock’s lower-frequency spectrum coincided with deeper surrounding water, whereas detection and repetition rates followed distinct site patterns. Shared structure and local variation provide a quantitative basis for cross-site identification and habitat monitoring that integrates call features, activity timing, and environmental conditions.

## Simple Summary

Fish sounds offer a way to study underwater activity in large, turbid rivers where direct observation is difficult. However, it remains unclear how much a fish call type varies between nearby habitats. We recorded fish sounds at four sites in the Cambodian Mekong River and compared their structure, timing, and repetition alongside local water depth, current speed, and background sound. The calls were attributed to the smallscale croaker using published descriptions and fish trapped at the study sites. Calls at all four sites shared a repeated-pulse structure and were concentrated in the late afternoon and early evening. Nevertheless, the distribution of sound energy and the speed of repeated calling differed among sites. Calls received at the deeper dock site had lower-frequency spectra, whereas calling sequences repeated more slowly at the eastern site. These findings show that a shared call pattern can coexist with local variation over short distances. Combining these features can help identify fish sounds and improve monitoring of fish activity and river habitats.

## 1. Introduction

Large tropical rivers sustain rich freshwater biodiversity and provide fisheries resources and food security for riverside communities [1,2]. River barriers, altered hydrological regimes, and habitat degradation are reshaping these ecosystems and increasing pressures on fish and other aquatic animals [3,4]. In the Mekong Basin, water infrastructure development and river fragmentation have affected fish migration routes and habitat connectivity [5]. Assessing fish responses to environmental change requires information not only on species composition and distribution, but also on activity timing, behavioral rhythms, and habitat use. Yet high turbidity, complex channels, and variable hydrological conditions make underwater behavior difficult to observe, and conventional capture and visual surveys rarely provide continuous records [6,7]. Long-term, minimally intrusive observations of fish activity are therefore important for understanding ecological processes in large tropical rivers.

Many fishes produce sounds during courtship, competition, defense, and other social interactions [8,9,10]. Passive acoustic monitoring (PAM) provides sustained records of natural sound production that complement capture and visual surveys [11]. FishSounds and global inventories of soniferous fishes have expanded the species information and reference recordings available for sound identification [12,13,14]. Applying these resources to ecological monitoring in a particular river, however, requires an understanding of natural variation in the target call type: which features remain similar across sites, and which vary with behavioral state, source location, or propagation conditions? Distinguishing relatively consistent structure from variable acoustic parameters is essential for identification across sites and interpretation of long-term recordings.

Sciaenids provide a useful group in which to examine this distinction. Their sonic muscle–swim bladder system typically produces low-frequency calls composed of repeated pulses, and vocal activity is closely associated with social and reproductive behavior [15]. The smallscale croaker (*Boesemania microlepis*) is a freshwater sciaenid of the Mekong Basin. Studies in southern Laos documented its use of deep-water habitats and calling aggregations associated with reproduction [16]. Mok et al. subsequently described calls recorded in aquaculture ponds, reservoirs, and rivers in Thailand, revealing multi-cycle pulses and prominent harmonic components [17]. These studies provide a reference for identifying *B. microlepis* calls in the wild. Research on the Amazonian silver croaker (*Plagioscion squamosissimus*) has also shown that freshwater sciaenid call structure and activity vary with hydrological stage, time, and location, highlighting the need to characterize natural variation beyond representative calls [18]. For *B. microlepis*, however, concurrent multisite comparisons within a natural river reach remain scarce. It is therefore unclear whether adjacent sites share a basic call structure and diel schedule, or how call spectra and sequence rhythms vary among them. Such comparisons can establish features suitable for identification across sites and reveal the spatial and temporal organization of calling in neighboring habitats, providing a basis for linking fish behavior to habitat conditions and using sound in sustained ecological monitoring. Mok et al. proposed that recoil of stretched aponeuroses connecting the sonic muscles to the swim bladder drives the multi-cycle pulses [17]. Regular pulse repetition can generate harmonics whose spacing reflects the pulse repetition period rather than the strongest spectral peak.

Interpreting spatial variation requires consideration of both calling behavior and recording conditions. Water depth, channel morphology, and flow can modify low-frequency propagation and received spectra, while background sound affects signal detectability [19,20,21]. Calling times, repetition rates, and sequence rhythms can also vary with behavioral state [22,23]. Multisite studies have documented spatial and temporal variation in fish acoustic activity associated with local habitat conditions [24,25]. Concurrent observations within a single reach reduce differences attributable to sampling dates; coupled with hydrological and background sound measurements, they can help identify environmental associations of acoustic variation among adjacent habitats.

The Kampi reach of the Cambodian Mekong River contains islands, narrow channels, and adjoining shallow and deep waters, creating pronounced environmental heterogeneity over short distances. We established four fixed acoustic monitoring sites across approximately 2 km of the reach and conducted concurrent monitoring over multiple days. These recordings were combined with fish occurrence records from November 2023, hydrological observations, and background sound measurements. We addressed three questions: (1) does the target low-frequency call type share repeated-pulse and harmonic structure across the four sites; (2) is calling concentrated within a common diel window; and (3) do call spectra and sequence rhythms differ among sites, and how are these differences related to depth, current speed, and background sound? The study provides a quantitative regional reference for identifying *B. microlepis* calls, investigating vocal behavior, and monitoring river habitats.

## 2. Materials and Methods

### 2.1. Study Area and Acoustic Monitoring

**Figure 1:**
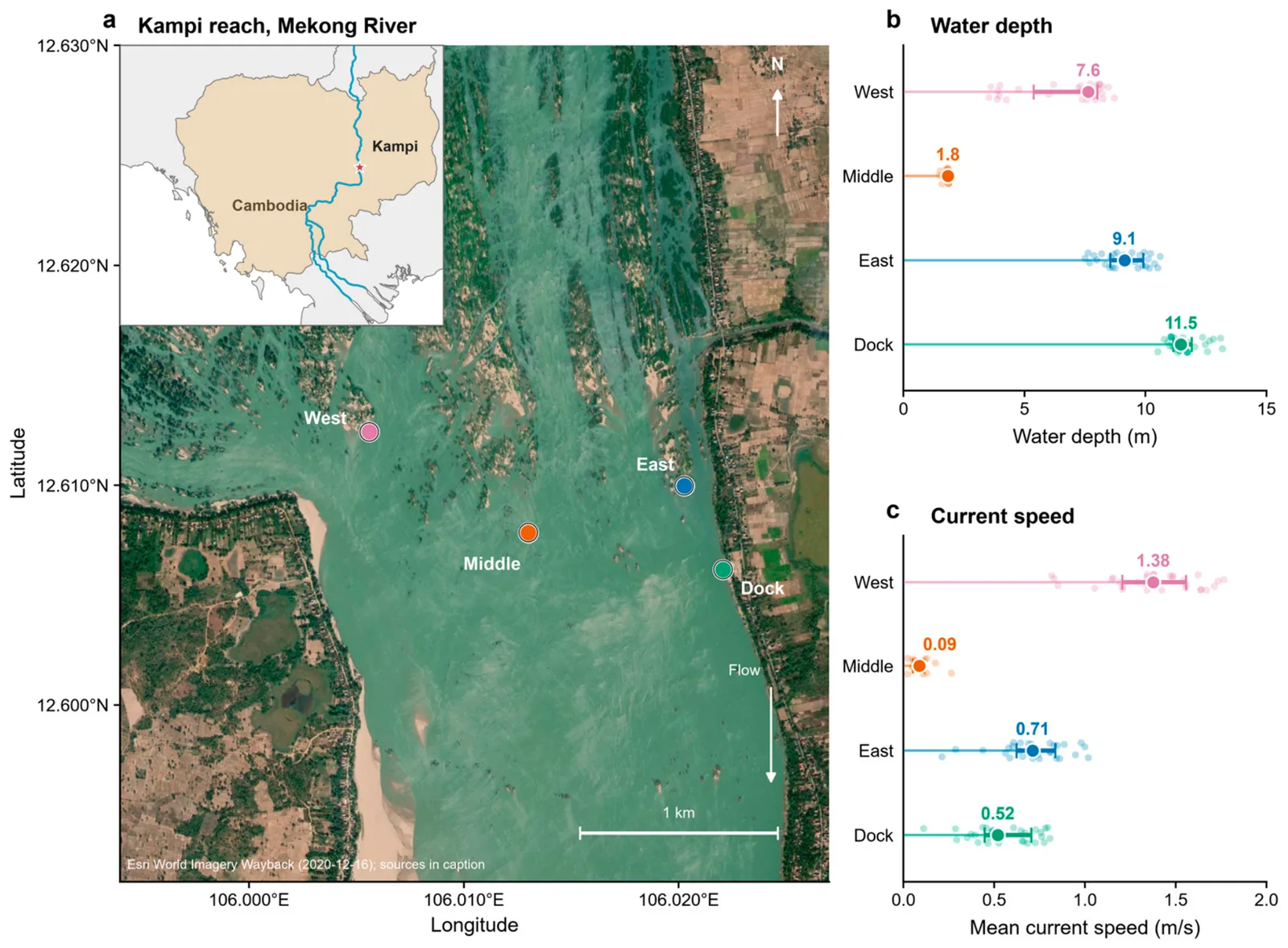
Acoustic monitoring sites and nearby hydrological measurements in the Kampi reach. (**a**) Locations of the four fixed monitoring sites; the red star in the regional inset indicates the Kampi study area. (**b**,**c**) Water depth and mean current speed measured by ADCP; small points represent valid ensembles, and large points and horizontal lines indicate medians and interquartile ranges. Satellite basemap: Esri World Imagery Wayback (16 December 2020 release). Sources: Esri, DigitalGlobe, Earthstar Geographics, CNES/Airbus DS, USDA FSA, USGS, Aerogrid, IGN, IGP, and the GIS User Community. The regional inset uses Natural Earth data.

Four fixed acoustic monitoring sites—West, Middle, East, and Dock—were established in the Kampi reach of the mainstem Mekong River in Kratie Province, Cambodia (Figure 1a). West was near islands and inter-island channels on the western side of the river, Middle was in the central channel, East was near the eastern bank, and Dock was adjacent to the landing on that bank.

Each site was equipped with a SoundTrap 300 HF autonomous underwater acoustic recorder that had been calibrated before deployment (Ocean Instruments, Auckland, New Zealand; manufacturer-confirmed sensitivity: −172 dB re 1 V/µPa) and sampled at 288 kHz. Recorders were secured to metal frames anchored to the riverbed, with the hydrophones approximately 1 m above the bed. The hydrophones were covered with stockings as improvised flow screens. Monitoring took place from 6 to 20 November 2023; all times are reported in Cambodian local time (UTC+7). West, Middle, and East recorded for 10 min every 30 min, whereas Dock recorded for 5 min every 10 min. Some recorders were retrieved early, leaving 9–19 November as the common analytical period across all four sites.

Water depth and current speed were measured along transects near the monitoring sites using an acoustic Doppler current profiler (ADCP; SonTek, San Diego, CA, USA) on 6 November 2023 (East and Dock), 9 November (Middle), and 10 November (West). Medians and first and third quartiles of valid ADCP ensembles were used to characterize the surrounding hydrological conditions (Figure 1b,c). Normalized waveform amplitudes were converted using the model-level high-gain full-scale pressure value of 172 dB re 1 µPa; no recorder-specific frequency-response correction was applied. Background noise in the concurrent recordings was quantified as the root-mean-square sound pressure level in the 180–3200 Hz band (dB re 1 µPa). These hydrological measurements were used for descriptive comparisons among sites, and background noise levels were used to examine associations with vocal features.

### 2.2. Detection of Low-Frequency Calls and Species Attribution

We developed a custom detection and interactive review program in Python 3.12 to process recordings from the four sites using pulse-train detection, spectral screening, and manual confirmation. The approach drew on feature-based detection of sciaenid calls [26] and manual annotation and verification methods [27]. Screening rules were refined through iterative visual and auditory review to accommodate nonstationary background sound, broadband transients, and weak signals before batch processing.

Recordings were first downsampled to 12 kHz with antialiasing and analyzed in 60 s blocks. A short-time Fourier transform with a 16 ms window and 4 ms hop provided time–frequency features over 100–4800 Hz for spectral screening. The Hilbert envelope of the waveform bandpass-filtered at 180–3200 Hz was used to locate pulses. To reduce the influence of nonstationary background sound, the smoothed envelope was robustly normalized against the local background:

(1) $$z(t)\;=\;\frac{e(t)\;-\;b(t)}{\max[1.4826m(t),\;\varepsilon ]}$$

where *z*(*t*) is the dimensionless normalized envelope and *e*(*t*) is the Hilbert envelope, low-pass filtered at 160 Hz and resampled to 1 kHz. The local background *b*(*t*) is the median of *e*(*t*) over a centered 1001-sample window (approximately 1 s), and *m*(*t*) is the median of |*e*(*t*) − *b*(*t*)| over the same window. The factor 1.4826 converts *m*(*t*) to a robust scale estimate. To prevent excessive amplification, ε is set to the larger of the analyzed block’s 10th percentile of |*e*(*t*) − *b*(*t*)| and 10^−8^ in waveform units.

Local peaks were then identified in the normalized envelope, and temporally adjacent peaks were grouped into candidate events. Screening used four feature groups: pulse rhythm, spectral coverage, energy persistence, and estimated signal-to-noise ratio (SNR). Pulse rhythm was characterized by pulse count, interpulse intervals, and their coefficient of variation. Spectral coverage was the proportion of frequency bins above the background threshold, and energy persistence was the proportion of low-frequency bins that remained above background during the candidate event. SNR was estimated as the difference between the 90th percentile of frame-level band-power levels within each 0.5 s analysis window and a local background baseline. Band power was averaged across 100–4800 Hz frequency bins and expressed in decibels. The baseline was the centered rolling 20th percentile of window-median band-power levels across 151 windows at 0.1 s steps (approximately 15 s). For candidate events spanning multiple 0.5 s windows, the maximum window-level estimate was assigned to the event. This estimate represented signal prominence relative to the local background. Additional searches recovered candidates with the target pulse structure but weak spectral features, as well as weak signals near repeated calling sequences. These candidates were reviewed together with the initial detections.

Candidates were inspected and auditioned using a Streamlit interface displaying waveforms and spectrograms alongside the audio. Two reviewers cross-checked candidates in the context of the original recordings, with particular attention to weak, overlapping, and contaminated signals; disagreements were resolved jointly. Both reviewers also examined the complete original recordings, including intervals without automatic detections, using waveforms and spectrograms; manually confirmed missed calls were added to the final event set. Detector recall was calculated as the proportion of calls in the final manually confirmed event set that had been detected automatically. Confirmed events were assigned onset and offset times, and duplicate detections were removed using the source file and temporal position. All confirmed events were used to analyze calling times. Calls with estimated SNR > 20 dB, clearly defined boundaries, and adequate quality for the relevant measurements were further selected for acoustic parameter analysis. This SNR threshold was not applied to the calling-time analysis.

In addition to the complete-recording review, both reviewers independently classified a blinded, site-stratified subset of 200 candidate events (50 per site, equally divided between previously retained and rejected candidates) as target or non-target for an agreement assessment; observed agreement and Cohen’s κ were calculated, with a 95% confidence interval obtained from 20,000 bootstrap resamples.

**Figure 2:**
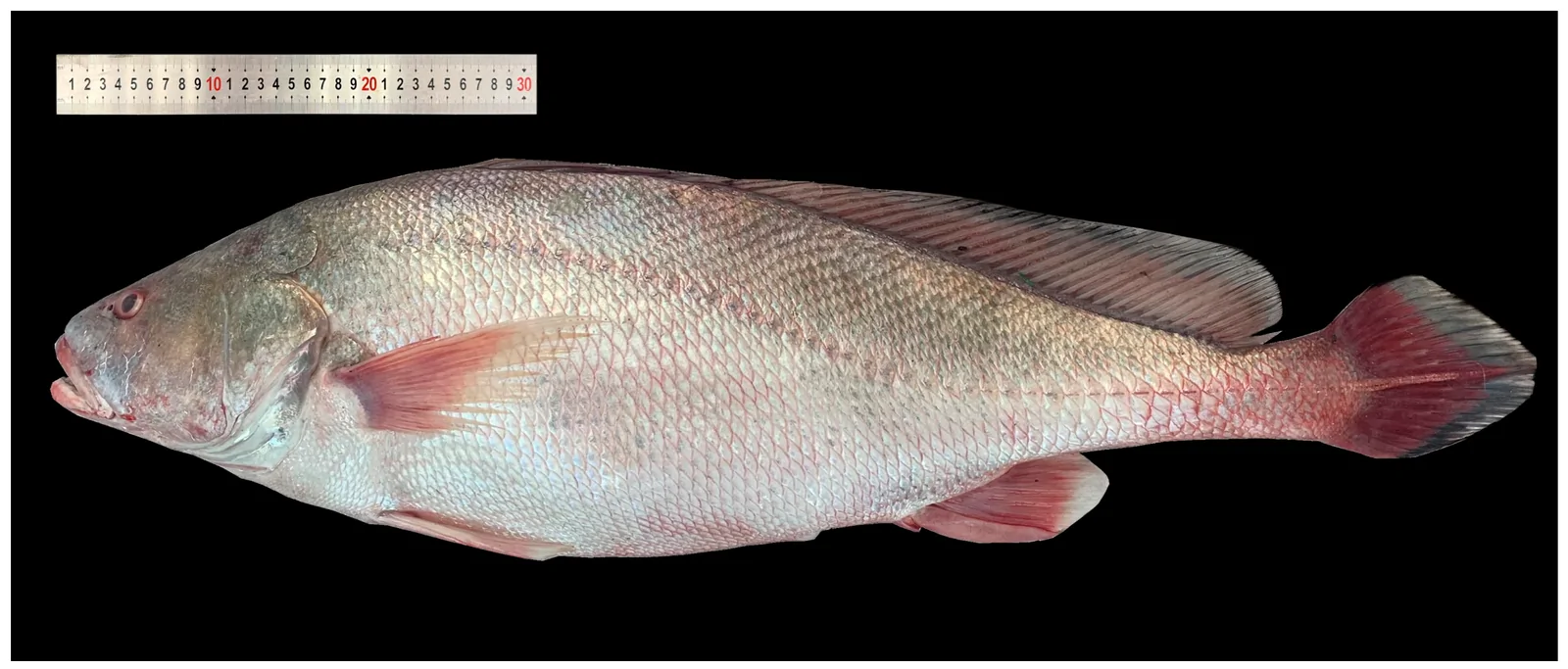
A *Boesemania microlepis* specimen captured during fish sampling in November 2023.

During the regional fish survey conducted near the four acoustic monitoring sites in November 2023, the research team used fish traps and also examined and morphologically identified local fishers’ net catches, recording 36 fish taxa (Table S1). Candidate sound sources included *B. microlepis*, *Hemibagrus spilopterus*, *Mystus bocourti*, and *M. singaringan*. The research team’s fish traps at West, Middle, East, and Dock yielded 2, 2, 1, and 2 morphologically identified *B. microlepis*, respectively, totaling 7 fish. Figure 2 shows a captured specimen. The detected signals were compared with available published sound descriptions for these candidates, using information from congeners where species-specific descriptions were lacking [28,29] and considering additional sound reports for fishes of the Mekong Basin [16]. The target calls exhibited regularly repeated multi-cycle tonal pulses, and their combined characteristics of duration, pulse periodicity, spectral distribution, and harmonic spacing were consistent with published descriptions of *B. microlepis* [16,17]. This acoustic comparison, together with capture evidence, supported attribution of the detected call type to *B. microlepis*.

### 2.3. Acoustic Characterization

Target vocalizations were characterized at the levels of individual calls and calling sequences [18,23], describing the temporal and spectral features of single calls and the temporal organization of successive calls, respectively.

Call duration *D* was defined as the interval between call onset and offset. To locate these boundaries, the waveform was downsampled to 48 kHz, filtered with a fourth-order, zero-phase Butterworth bandpass filter at 80–5000 Hz, and used to obtain a smoothed Hilbert envelope. An activity threshold was set at the local background median plus five robust standard deviations. Boundaries were estimated from the 1–99% cumulative interval of the squared suprathreshold envelope and then checked against the waveform and spectrogram.

These spectral parameters were derived from the received signals without transmission-loss or time-varying-gain correction because source–receiver distances were unknown. Peak frequency *f_p_* and the 95% cumulative-energy frequency *f*_95_ described the distribution of spectral energy within the 180–5000 Hz analysis band. Power spectral densities (PSDs) of calls and nearby segments without target calls were estimated using Welch’s method with Hann windows, segments of no more than 16,384 samples, and 75% overlap [30,31]. A common FFT length was used for call and background PSDs; where necessary, background PSDs were interpolated to the call frequency grid. The median PSD across background segments was taken at each frequency to obtain a background spectrum. The background-corrected spectrum was defined as:

*S*(*f*) = max[*P*_event_(*f*) − *P*_background_(*f*), 0] (2)

where *P*_event_(*f*) and *P*_background_(*f*) are the call and background PSDs, respectively. Peak frequency *f_p_* was the frequency at the maximum of *S*(*f*) after smoothing over approximately 20 Hz. The 95% cumulative-energy frequency *f*_95_ was calculated from the unsmoothed *S*(*f*) as the lowest frequency at which cumulative energy reached 95% of the total within the analysis band:

(3) $$\frac{\int_{180}^{f95}S(f)\;\mathrm{df}}{\int_{180}^{5000}S(f)\;\mathrm{df}}\geq 0.95$$

Pulse peaks were identified on the smoothed Hilbert envelope (low-pass filtered at 180 Hz and resampled to 2 kHz) of the waveform bandpass-filtered at 180–3200 Hz, using an adaptive threshold based on the adjacent background. Peak times were refined on a 48 kHz version of the same smoothed envelope by locating the maximum within ±0.5 ms of each initial peak and applying three-point parabolic interpolation. Pulse rate *r_p_* within a call was calculated as the reciprocal of the median interval between successive pulse peaks, *r_p_* = 1/median(*t_i_*_+1_ − *t_i_*), where *t_i_* is measured in seconds and indexes successive pulse peaks. Only calls with clearly resolved pulse peaks in the waveform and envelope were used. Pulse measurability was reviewed for the original 572 calls: 132 calls with ambiguous recurrent weak peaks or mixed pulse organization were excluded only from pulse-rate analysis, leaving 440 eligible calls. Pulse rate and harmonic spacing were compared within the same calls.

For calls with clearly resolved spectral lines, harmonic spacing Δ*f_h_* and maximum sustained frequency *f*_max_ were also calculated to describe spectral periodicity and high-frequency extension. Harmonic spacing was estimated by fitting stable peaks between 80 and 5000 Hz to integer harmonic orders. Maximum sustained frequency was estimated from a separate 48 kHz waveform without the 80–5000 Hz filter used for boundary estimation, with an upper search limit of 20 kHz. It was defined as the upper edge of the highest qualifying band that exceeded the adjacent background by at least 6 dB, persisted over time, and co-occurred with the low-frequency core of the call.

Sequences were analyzed as observed runs within individual WAV files. Calls were not joined across recording gaps, and unrecorded intervals did not enter the sequence metrics. Calling sequences comprised successive calls with similar acoustic morphology. Manual checks removed clearly overlapping sequences and nested duplicates. The median onset-to-onset interval *I*_50_, within-sequence call repetition rate *R*, and coefficient of variation in the intervals *CV_I_* described the typical spacing between successive calls, their repetition speed, and the relative dispersion of intervals. For a sequence of *n* calls with onset times *t*_1_, …, *t_n_*, successive onset intervals *I_i_*, *CV_I_*, and *R* were calculated as follows:

(4) $$I_{i}\;=\;t_{i+1}\;-\;t_{i},{\mathrm{CV}}_{I}\;=\;\frac{s_{I}}{\bar{I}},R\;=\;\frac{n\;-\;1}{t_{n}\;-\;t_{1}}$$

where *i* = 1, …, *n* − 1, and $\bar{I}$ and *s_I_* are the mean and sample standard deviation of the onset intervals within a sequence. *I*_50_ is the median of *I_i_*. Each sequence was represented by its values of *I*_50_, *CV_I_*, and *R* in subsequent analyses.

### 2.4. Calling Patterns and Statistical Analysis

Diel calling patterns were described by pooling events into hourly bins and expressing the count in each bin as a percentage of all events recorded at that site:

(5) $$p_{s,h}\;=\;\frac{N_{s,h}}{\sum_{h=0}^{23}N_{s,h}}\times 100\%$$

where *p_s_*_,*h*_ is the percentage of events at site *s* in hourly bin *h*, and *N_s_*_,*h*_ is the event count in that bin pooled across all included dates. This summary uses the site-specific full-recording scope. For the 11 dates covered by all four sites, we also calculated the daily percentage of events occurring from 16:00 to 20:00 to compare late-afternoon and early-evening concentration. Overall site-level detection rates were calculated by dividing confirmed event counts by the actual number of valid recording minutes within 239 hourly periods with recordings at all four sites.

Acoustic parameters were summarized using medians and first and third quartiles. To account for the non-independence of calls within recordings and dates [32], a daily median was first calculated for each parameter at each site. Site comparisons then treated dates with data at all four sites as matched blocks. Friedman tests were applied to call duration, peak frequency, 95% cumulative-energy frequency, pulse rate, and the three sequence parameters. Kendall’s *W* was used as the overall effect size, and the *p* values from the seven tests were adjusted using the Benjamini–Hochberg procedure [33].

Pairwise differences were assessed using two-sided paired Wilcoxon signed-rank tests, with Holm adjustment across the six site pairs for each parameter [34]. Pairwise results were interpreted only when the corresponding overall test yielded an adjusted *q* < 0.05. The difference between two sites was expressed as the median of the date-specific differences between their daily parameter medians. A 95% confidence interval (CI) was estimated from 20,000 bootstrap resamples at the date level. As a sensitivity analysis, Kruskal–Wallis tests were applied to daily medians from all available dates at each site, again with Benjamini–Hochberg adjustment across the seven tests, to assess consistency across date ranges and analytical approaches. Sequence sensitivity checks excluded runs within 1 s of a file edge and, separately, retained runs wholly inside common 5 min analysis blocks with a 1 s edge margin. Cross-site timing checks across all six site pairs used separate recording subsets for alignment estimation and evaluation, with within-recording time shifts as coincidence controls. Selected candidate matches were assessed using raw waveform, envelope and spectral comparisons against different-date calls.

Background sound was extracted from unclipped 5 s segments that did not overlap confirmed calls or the 1 s intervals preceding and following them. Each hour was required to contain at least 60 s of usable background and at least 10% of its valid recording duration. Within each eligible hour, three 5 s segments were selected at approximately the one-sixth, one-half, and five-sixths positions in the chronologically ordered list of usable segments. PSDs were estimated using Welch’s method with 1 s Hann windows and 50% overlap, then integrated over 180–3200 Hz. Powers from three segments were averaged and converted to the hourly background sound level. Environmental associations were assessed using ordinary least-squares regression. Hourly detection rates were transformed as log(1 + detection rate). Pooled models included categorical site, hour-of-day, and date effects, together with site-by-hour interactions; site-specific models included hour-of-day and date effects. For acoustic parameters, matched observations were summarized first by hour and then by site and date using medians. These models included categorical site and date effects and median sampling hour as a continuous covariate. Confidence intervals were estimated with clustering by date, and *p* values were obtained from a sign-flip test based on date-block scores. Benjamini–Hochberg adjustment was applied separately to 30 detection-rate tests and 19 acoustic-parameter tests. The 30 tests crossed five sound metrics with six subsets: pooled observations, each site, and pooled observations at 16:00–20:00. The metrics were overall RMS level, the level exceeded 95% of the time, call-excluded median level, and background levels at 180–3200 and 180–5000 Hz. The 19 tests comprised the seven primary acoustic parameters against overall RMS and call-excluded median levels, plus five parameters against 180–3200 Hz background level. These five were peak frequency, 95% cumulative-energy frequency, and the three sequence parameters. All analyses were conducted in Python using NumPy, pandas, SciPy [35], and statsmodels. Target-call exclusion did not establish that other biological sounds were absent, and vessel-noise periods were not separately labelled or removed.

## 3. Results

### 3.1. Basic Structure of Low-Frequency Calls

**Figure 3:**
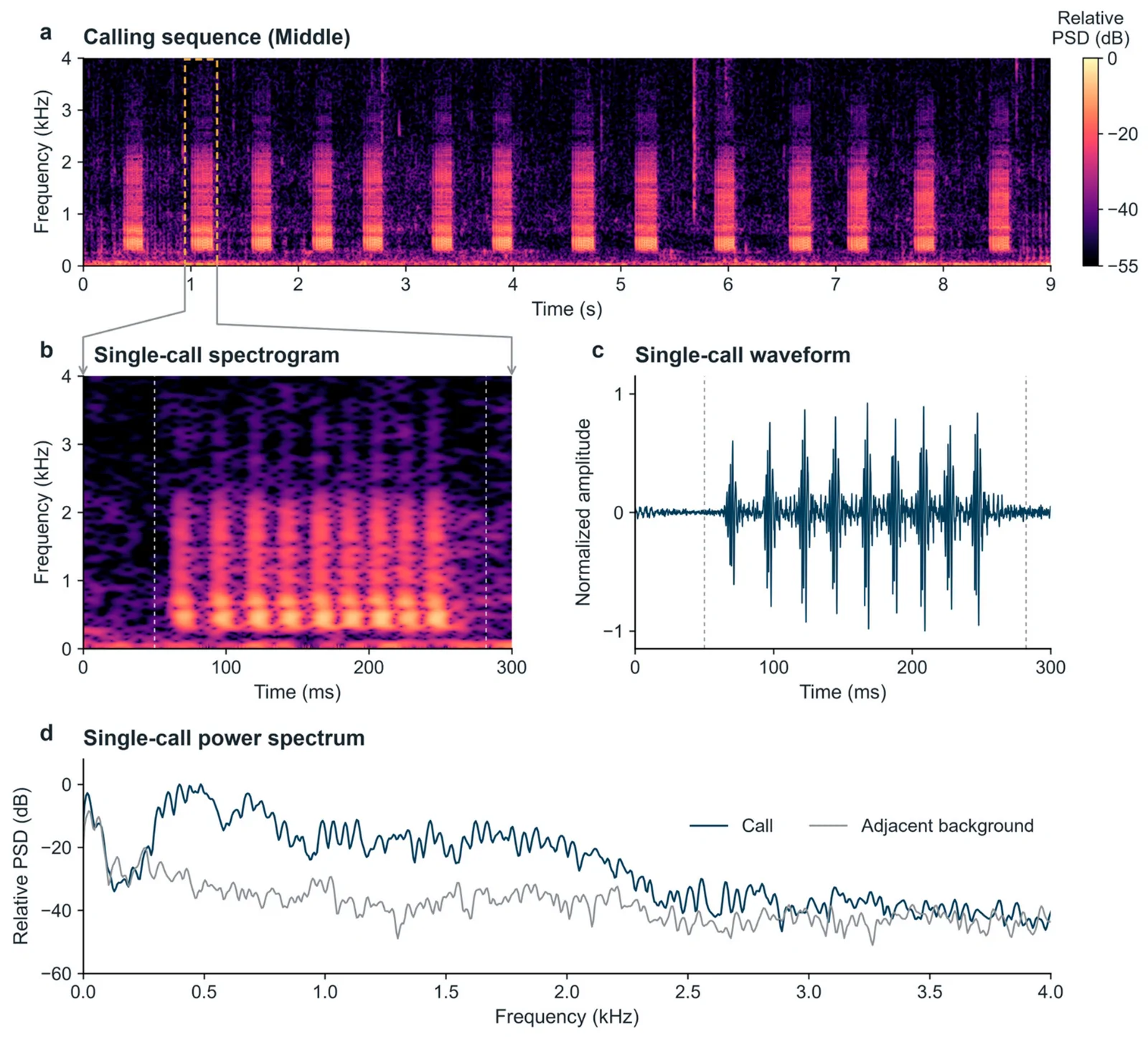
Calling sequence and individual-call structure of the target low-frequency fish sound, illustrated with a continuous recording from Middle. (**a**) Spectrogram of a sequence containing 14 calls; the dashed rectangle marks the selected call, and gray arrows link it to the enlarged view in (**b**). (**b**) Spectrogram of that call. Vertical dashed lines in (**b**,**c**) mark the annotated onset and offset of the selected call. (**c**) Its waveform after 180–3200 Hz bandpass filtering and amplitude normalization. (**d**) Power spectra of the call and an adjacent segment without target calls. Spectrograms in (**a**,**b**) used 42.67 and 21.33 ms Hann windows, 8192- and 4096-point FFTs, and hops of 4 and 0.5 ms, respectively. In (**d**), both spectra are expressed relative to a common reference; the background spectrum was not subtracted from the call spectrum.

Target calls were brief and composed of repeated pulses; successive calls were separated by intervals to form calling sequences (Figure 3a). Pulse-energy bands in the spectrograms corresponded to pulse oscillations in the waveforms (Figure 3b,c), and power in the main frequency band exceeded that of the adjacent background (Figure 3d).

Site medians of call duration and pulse rate ranged from 0.172 to 0.187 s and from 112.5 to 115.5 pulses/s, respectively (Table 1). Median peak frequency ranged from 452.6 to 679.0 Hz, and median 95% cumulative-energy frequency from 1145.9 to 1423.8 Hz, indicating that energy was concentrated at low frequencies. For calls with clearly resolved spectral lines, site-median harmonic spacing ranged from 114.8 to 119.1 Hz. The median absolute difference was 0.7% of harmonic spacing in the 70 calls with both measurements. Some calls also showed sustained high-frequency components, with maximum sustained frequencies across all valid observations ranging from 2.03 to 5.21 kHz.

### 3.2. Spatial and Diel Patterns of Calling Activity

Across the complete reviewed recordings, detector recall before manual recovery was 95.83%, 94.83%, 93.87%, and 95.18% at West, Middle, East, and Dock, respectively. The two reviewers agreed on 188 of the 200 independently classified candidate events (94.0% agreement; Cohen’s κ = 0.88; bootstrap 95% CI 0.81–0.94). Across the 239 hourly periods with recordings at all four sites during 9–19 November 2023, West, Middle, East, and Dock yielded 28,740, 23,762, 11,002, and 26,499 confirmed events, respectively. Rates based on valid recording duration were 6.06, 5.01, 2.32, and 3.76 calls/min, respectively. In a separate cross-site timing check, excess coincidence between East and Dock was 5.65% of East events and 3.88% of Dock events; date-bootstrap intervals for the other five site pairs included zero. These estimates were conditional on the acoustic alignment and onset-matching criteria. These descriptive rates characterize received events; detection probability was not assumed equal among sites.

**Figure 4:**
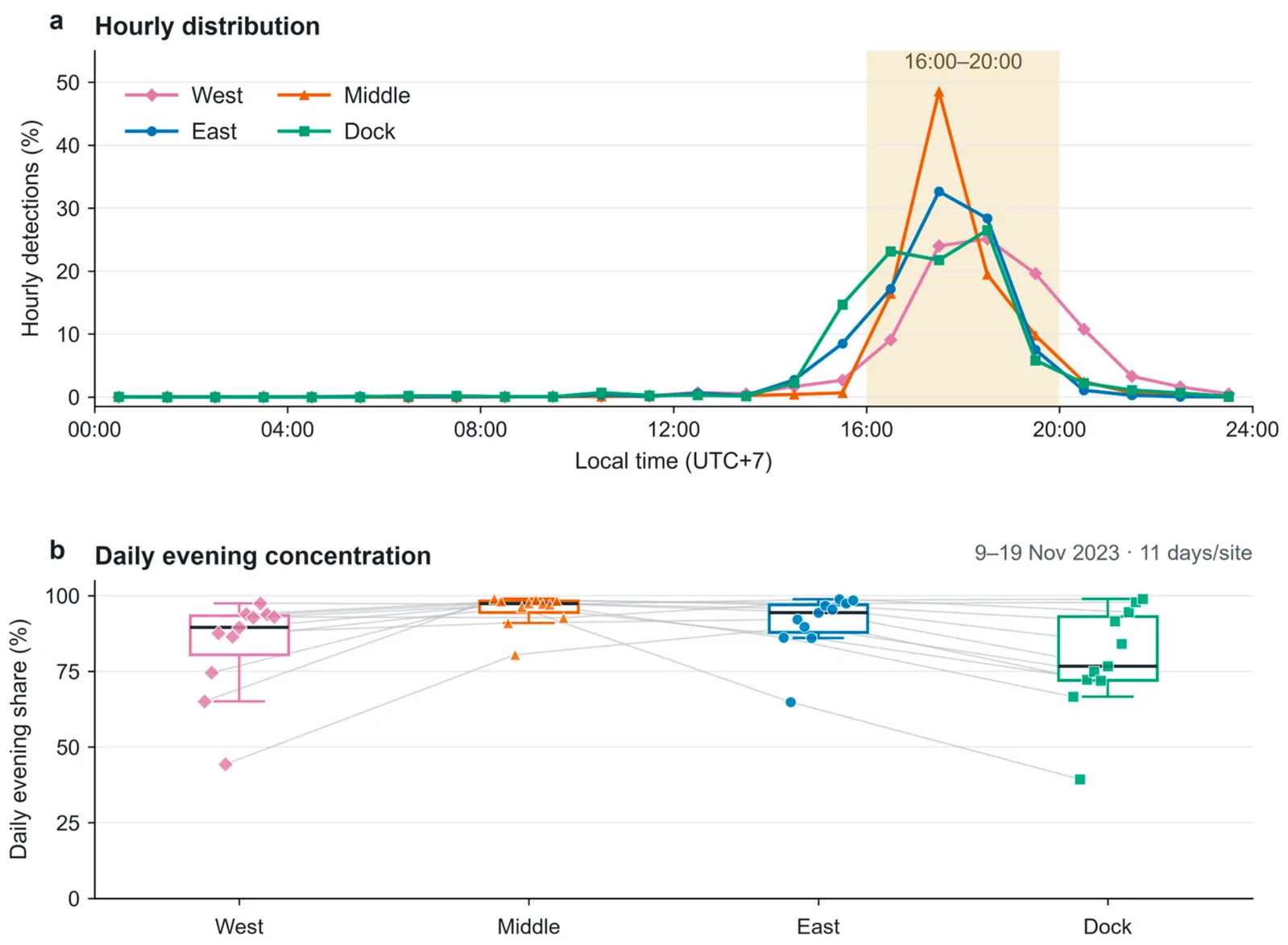
Diel distribution of low-frequency calls and their concentration from late afternoon to early evening. (**a**) Within-site event percentages in 1 h bins, plotted at bin midpoints; shading marks 16:00–20:00. (**b**) Daily percentage of events occurring from 16:00 to 20:00 at each site on 9–19 November 2023. Each point represents one date, with gray lines connecting the same date across sites. Boxes span the 25th–75th percentiles, central lines show medians, and whiskers extend to the most extreme observations within 1.5 interquartile ranges of the box. All observations are shown. Pink diamonds, orange triangles, blue circles, and green squares represent West, Middle, East, and Dock, respectively. Times are Cambodian local time (UTC+7), and daily percentages are calculated by local calendar date.

Calling was concentrated from 16:00 to 20:00 at all four sites. This interval accounted for 77.8%, 94.2%, 85.7%, and 77.3% of events at West, Middle, East, and Dock, respectively (Figure 4a). Hourly event percentages peaked at 17:00–18:00 at Middle and East and at 18:00–19:00 at West and Dock, with more concentrated diel distributions at the former two sites. Daily summaries showed the same pattern (Figure 4b). Median daily percentages of events in this interval [first, third quartiles] were 89.4% [80.5%, 93.4%], 97.4% [94.5%, 98.3%], 94.4% [87.9%, 97.1%], and 76.7% [72.1%, 93.1%] at the four sites, respectively.

### 3.3. Site Differences in Acoustic Parameters

**Figure 5:**
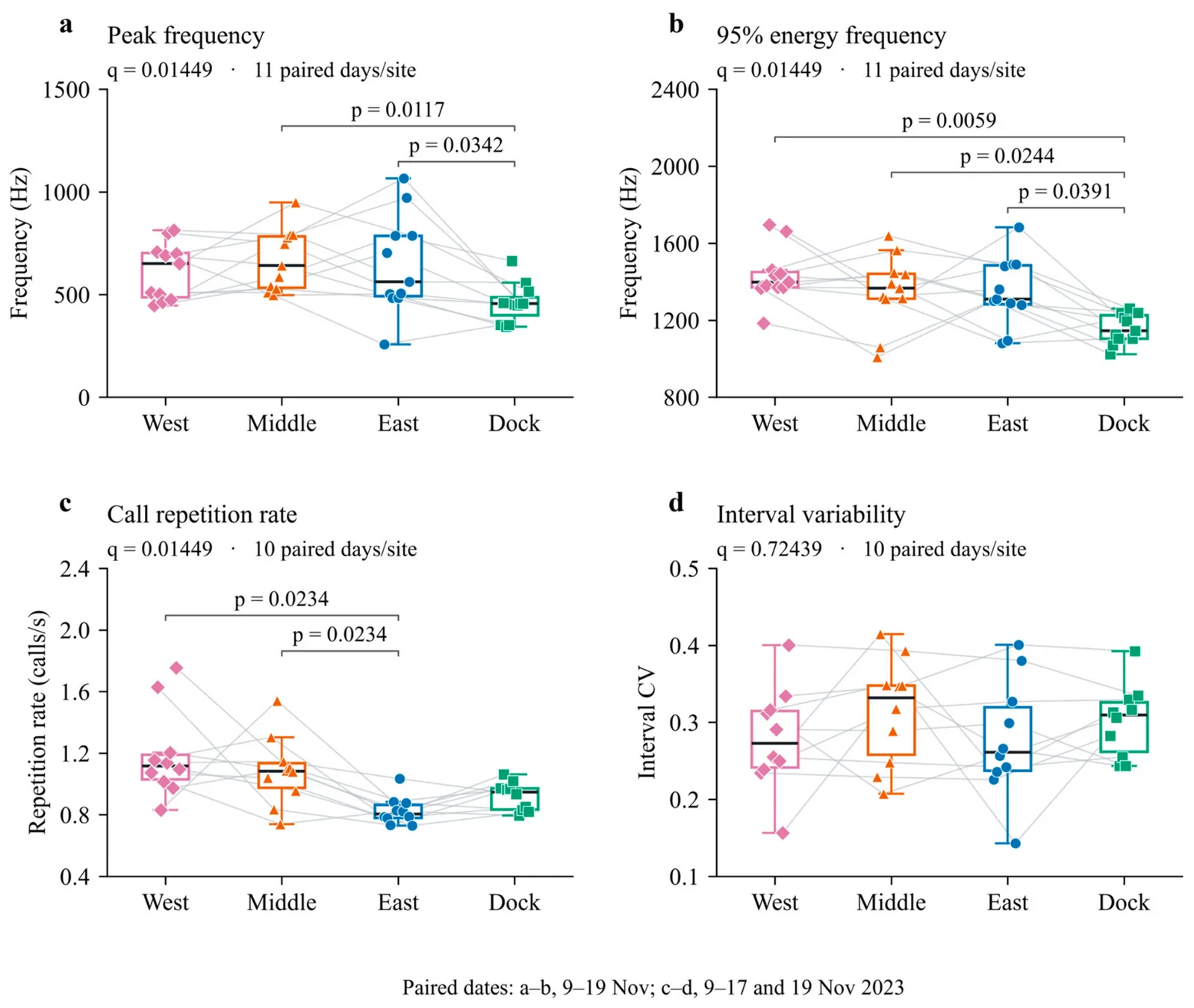
Spectral features and temporal organization of calling sequences at the four sites. (**a**) Peak frequency. (**b**) 95% cumulative-energy frequency. (**c**) Call repetition rate within sequences. (**d**) Coefficient of variation in onset intervals. Points represent daily site medians, with gray lines connecting the same date across sites. Pink diamonds, orange triangles, blue circles, and green squares represent West, Middle, East, and Dock, respectively. Panels (**a**,**b**) include 11 matched dates per site, and panels (**c**,**d**) include 10. Boxplot elements are defined as in Figure 4b. The *q* values are Benjamini–Hochberg-adjusted results from Friedman tests of the seven primary parameters. *p* values above brackets are Holm-adjusted results from the six paired Wilcoxon comparisons within each parameter.

Spectral features differed significantly among sites. Overall tests were significant for both peak frequency and 95% cumulative-energy frequency (*q* = 0.014), with Kendall’s *W* values of 0.356 and 0.372, respectively (Figure 5a,b). Peak frequency at Dock was lower than at Middle and East, with median paired differences of −189.0 and −131.8 Hz (Holm-adjusted *p* = 0.012 and 0.034, respectively). The 95% cumulative-energy frequency at Dock was also lower than at the other three sites, with median paired differences of −221.9, −200.0, and −235.1 Hz relative to West, Middle, and East, respectively (all Holm-adjusted *p* < 0.05). Together, these metrics indicate a shift toward lower frequencies in the spectral energy distribution of calls recorded at Dock. Call duration and pulse rate did not differ significantly among sites in the matched-date analysis (*q* = 0.161 and 0.067, respectively).

Sequence-level differences primarily concerned call repetition speed. Overall tests were significant for both the median onset interval and call repetition rate (*q* = 0.014), with *W* values of 0.412 and 0.432, respectively. The median onset interval at East was 0.275 s longer than at West (95% CI: 0.086–0.490 s; Holm-adjusted *p* = 0.023). Call repetition rate at East was 0.327 and 0.241 calls/s lower than at West and Middle, respectively (both Holm-adjusted *p* = 0.023; Figure 5c). The coefficient of variation in onset intervals did not differ significantly among sites (*q* = 0.724, *W* = 0.044; Figure 5d).

Call duration differed among sites in the all-date sensitivity analysis but not in the matched-date analysis; overall significance conclusions agreed for the other six parameters. Excluding file-edge sequences and using equal 5 min blocks retained the lower repetition rate at East relative to West and Middle and the nonsignificant site difference in interval variability.

**Figure 6:**
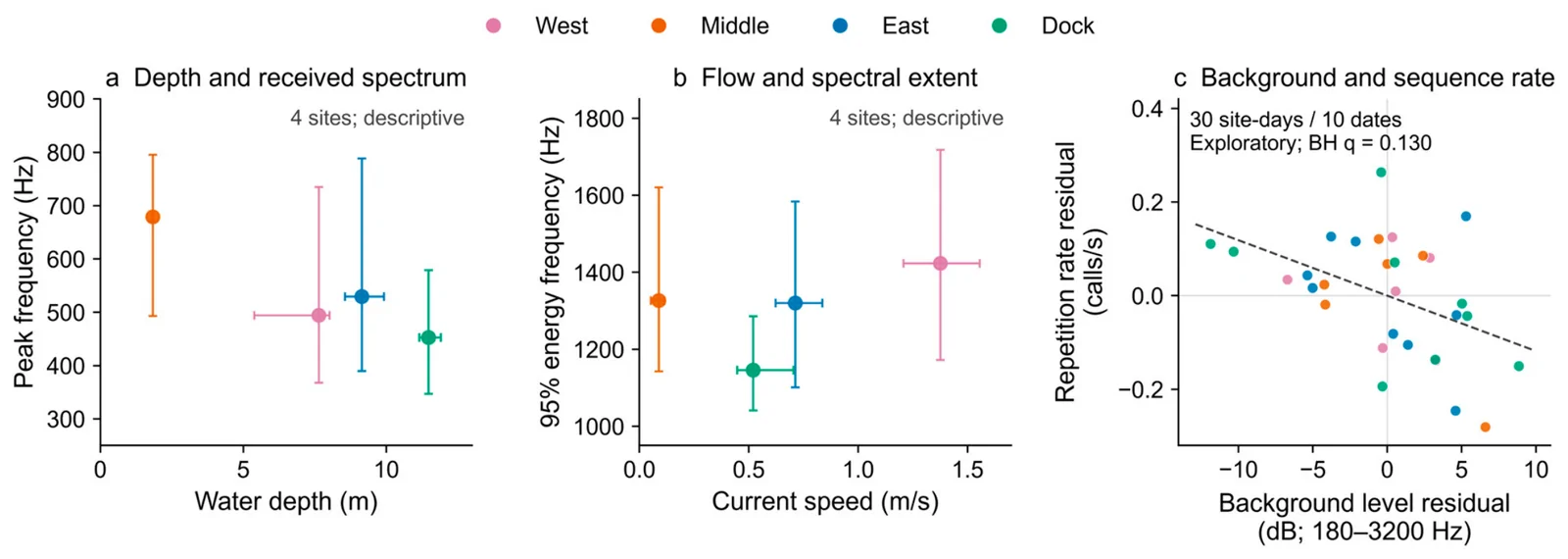
Comparisons between low-frequency call features and environmental conditions. (**a**) Water depth and peak frequency. (**b**) Current speed and 95% cumulative-energy frequency. Each point in (**a**,**b**) represents one site; horizontal and vertical bars span the first to third quartiles of hydrological and acoustic measurements, respectively, using data from Figure 1 and Table 1. (**c**) Partial regression plot of background sound level and within-sequence call repetition rate after adjustment for site, date, and sampling time. Points represent 30 eligible site–date observations with matched background and sequence data (West 6, Middle 6, East 9, and Dock 9), spanning 10 dates. Axes show residuals after removing the covariate effects, and the dashed line represents the fitted relationship. The *q* value was obtained by Benjamini–Hochberg adjustment across 19 acoustic-parameter association tests. Panels (**a**,**b**) are descriptive four-site comparisons; panel (**c**) shows an exploratory association.

After adjustment for site, date, and sampling time, background sound level in the 180–3200 Hz band was not significantly associated with detection rate, peak frequency, or 95% cumulative-energy frequency after multiple-testing correction. Its association with call repetition rate was negative but also nonsignificant after correction (*q* = 0.130; Figure 6c).

## 4. Discussion

### 4.1. Shared Call Structure and Natural Variation

Concurrent recordings at the four Kampi sites revealed a shared pulsed and harmonic call structure alongside differences in received spectral energy distribution and within-sequence repetition rates. These observations extend earlier ecological accounts and representative descriptions of *B. microlepis* [16,17] to spatial comparisons within a natural river reach. Identification in the wild requires both a representative call description and an understanding of parameter variation under different recording conditions.

Agreement between pulse rate and harmonic spacing within the same 70 calls supports a common pulse-periodicity basis. In this subset, site-median pulse rates of 114.1–118.8 pulses/s were comparable to the approximately 104–118 Hz cepstral fundamental frequencies reported by Mok et al. [17]. Pulse rates showed no significant site difference in the primary analysis. Together with multi-cycle pulse structure and local capture evidence, this agreement supports attribution to *B. microlepis*, although individual callers were not directly identified.

Peak frequency showed more pronounced site differences than harmonic spacing. Changes in the relative energy of harmonics can shift the dominant spectral peak without changing pulse periodicity. Mok et al. also observed differences in the main energy bands of *B. microlepis* calls among recording environments [17]. At Dock, both peak frequency and 95% cumulative-energy frequency were lower, indicating energy redistribution across a broader band rather than a change in one spectral peak.

Bolgan et al. found that *Argyrosomus regius* calls recorded across environments shared basic pulse structure but varied substantially in pulse number [36]. Several predefined call categories also overlapped in multivariate analyses, indicating that individual parameter differences need not define distinct call types. Combining pulse organization and spectral periodicity with observed parameter ranges therefore provides a more informative identification reference than isolated frequency peaks.

### 4.2. Diel Concentration and Temporal Organization Across Scales

Calls were concentrated at 16:00–20:00 local time across all four sites, and daily summaries showed that this pattern recurred during the common monitoring period. The window spans sunset, while earlier peaks at Middle and East indicate local differences within a shared schedule. Individual-call structure was not compared among diel periods, so the hourly distributions do not establish structural changes through the day.

Evening calling has been documented for *B. microlepis* in captivity [17], and Kim et al. observed concentration around sunset and twilight in cultured *Miichthys miiuy* [37]. These findings suggest that changes in light may organize sciaenid calling schedules, while local activity and social interactions could modify peak timing and concentration. The shared window and different peak times at Kampi are consistent with common temporal cues acting alongside local behavioral variation.

Repeated sequences of brief calls are consistent with sustained social communication. Calling activity and temporal features vary with reproductive condition and spawning in cultured sciaenids [38]. However, Amazonian silver croaker also produce advertisement calls outside the reproductive season [18], showing that regular calling is not exclusive to spawning. The Kampi recordings therefore support a social communication interpretation but do not establish a spawning aggregation. Gonadal state and reproductive behavior were not monitored, so call changes during the reproductive process could not be assessed.

Diel concentration and call repetition within sequences describe distinct levels of temporal organization. Middle combined a concentrated diel distribution with relatively rapid call repetition, whereas East combined concentrated activity with slower repetition. The former describes how calls are distributed over a day; the latter describes the spacing of successive calls within a sequence.

### 4.3. Environmental and Behavioral Explanations for Site Differences

Spectra, detection rates, and within-sequence call repetition did not follow identical spatial patterns, suggesting that no single environmental gradient accounts for all variation. Interpretation therefore requires distinguishing biological source variation, frequency-dependent propagation, and detectability. Depth, channel form, and flow can influence both habitat use and call transmission to a recorder. The four-site comparisons in Figure 6a,b are descriptive and do not establish depth or current speed as determinants of call structure.

Dock’s lower-frequency received spectra coincided with deeper surrounding water, approximately 11.5 m compared with 1.8 m near Middle (Figure 1 and Figure 6a). Shallow-water experiments [20] and river transmission studies [39] show that depth and channel conditions can limit low-frequency propagation. Greater retention of low-frequency components along deeper paths is therefore a plausible explanation for the received spectral shift at Dock. Source positions and distances were unknown, and no transmission-loss or time-varying-gain correction was applied. The pattern consequently does not distinguish propagation filtering from changes in emitted calls. Background subtraction does not reverse frequency-dependent transmission effects in the received spectrum.

Depth alone does not explain the full spectral pattern. Middle had the highest peak frequency, whereas West had the highest 95% cumulative-energy frequency. Island channels near West may provide propagation paths and source positions different from open shallow water or deep near-bank habitat. River soundscapes vary with roughness and depth–velocity combinations [19], while riverbed properties and bubble processes may affect attenuation [39]. Fish size and water temperature can also alter sciaenid call parameters [40], indicating that source characteristics and propagation may jointly shape received spectra.

Confirmed received-event rates were higher at West and Middle and lowest at East after recording-time normalization. West and Middle nevertheless differed in flow and typical background levels, approximately 96.78 and 89.25 dB, respectively. A stronger background therefore did not necessarily correspond to lower recorded activity. Local calling activity, source distance, and propagation paths may contribute to this pattern. Detection rates consequently reflect both vocal activity and detection conditions and cannot be equated directly with fish abundance.

Adjusted models did not support associations of background level with detection rate or spectral parameters. Auditory masking in fishes depends on species and frequency [41], so equal overall sound levels need not imply equivalent communication or detection conditions. Flow-induced pseudo-noise and vessel contributions were not measured separately. These analyses therefore concern aggregate background sound rather than isolated boat or flow effects.

Within-sequence call repetition was slower at East than at West and Middle, whereas interval variability showed no significant site difference. Multisite studies of brown meagre likewise show that shared rhythmic organization can coexist with local differences in repetition speed [23]. Local social context and calling motivation are plausible behavioral explanations for these differences. The principal East contrasts persisted after excluding file-edge runs and using equal 5 min analysis blocks, indicating that recording-window differences do not fully account for the pattern.

The negative background–repetition association was nonsignificant after adjustment (*q* = 0.130; Figure 6c), so these data do not establish noise-induced slowing. Repeated boat-noise exposure in brown meagre was followed by an increase in recorded pulse counts [42], illustrating that count-based activity and within-sequence timing may respond differently. Environmental analyses should therefore distinguish call counts, sequence rhythms, and received spectra rather than treat them as equivalent measures of activity.

### 4.4. Monitoring Implications and Study Limitations

PAM has helped fill gaps in the distribution and conservation status of inconspicuous fishes [43], but management applications require alignment of source knowledge, sampling design, and interpretation [44]. The Kampi structural reference and evening calling concentration can inform call identification and recording schedules. Sampling island channels, shallow waters, and deep near-bank habitats would support both habitat coverage and comparability among receiving conditions. Increasing recording effort during peak hours while retaining whole-day coverage would balance efficiency and temporal representativeness [45].

Sampling covered a single November period, with 11 common dates and one recorder per site. Repeated dates support comparisons among these receiving locations, but site, recorder, and habitat effects remain confounded. Fish may move among nearby sites. Cross-site timing and raw-audio comparisons supported partial shared reception between East and Dock, but the excess coincidence estimates were conditional on acoustic alignment and matching criteria. The design therefore does not establish separate populations or independent callers at the four sites. Regional *B. microlepis* accounts link vocal aggregations to seasonal reproduction [16], and silver croaker calls vary with hydrological stage [18]. The observed call characteristics and diel schedule should therefore be tested across seasons before being generalized beyond the sampling period.

Manual review and recovery of missed calls do not establish equal acoustic detectability among sites. High-SNR selection favors conspicuous received signals, while duty-cycled recordings sample observed sequences rather than complete calling bouts. Consistent processing and linked audio recordings, species identifications, and environmental metadata would improve comparisons across sites and periods [46]. Controlled-source propagation measurements and acoustic localization combined with imaging [47] would help separate transmission effects from source behavior.

## 5. Conclusions

Concurrent recordings at four sites in the Kampi reach revealed shared pulsed and harmonic structure in low-frequency calls attributed to *B. microlepis*. Calling was concentrated at 16:00–20:00 local time, while received spectra, within-sequence repetition, and event rates normalized by valid recording duration showed different spatial patterns. Lower-frequency spectra at Dock coincided with deeper surrounding water, whereas East had slower within-sequence repetition than West and Middle.

These observations document acoustic heterogeneity within one river reach and highlight the need to consider biological source variation, sound propagation, and detectability. By extending earlier regional call descriptions to spatial and temporal comparisons, this study provides a quantitative reference for received call variation in a natural river. This reference can inform regional call identification, acoustic sampling design, and monitoring of fish vocal activity.

## Figures and Tables

**Table 1:** Acoustic parameters of low-frequency calls at the four sites.

Parameter	West	Middle	East	Dock
Call duration *D* (s)	0.179 [0.160, 0.201] *n* = 193	0.185 [0.162, 0.207] *n* = 193	0.172 [0.149, 0.189] *n* = 91	0.187 [0.163, 0.209] *n* = 255
Peak frequency *f_p_* (Hz)	494.4 [368.0, 735.0] *n* = 192	679.0 [493.3, 795.4] *n* = 182	529.5 [390.0, 788.8] *n* = 72	452.6 [347.2, 579.0] *n* = 126
95% cumulative-energy frequency *f*_95_ (Hz)	1423.8 [1172.2, 1718.3] *n* = 192	1327.1 [1142.6, 1620.5] *n* = 182	1320.6 [1101.4, 1584.2] *n* = 72	1145.9 [1041.5, 1285.9] *n* = 126
Pulse rate *r_p_* (pulses/s)	113.2 [47.2, 116.8] *n* = 105	112.5 [93.9, 115.5] *n* = 147	115.5 [111.8, 119.5] *n* = 62	112.7 [109.9, 116.4] *n* = 126
Median onset interval *I*_50_ (s)	0.849 [0.651, 1.336] *n* = 55	0.903 [0.780, 1.066] *n* = 59	1.156 [0.994, 1.325] *n* = 38	1.144 [0.998, 1.246] *n* = 59
Call repetition rate *R* (calls/s)	1.073 [0.736, 1.412] *n* = 55	1.096 [0.948, 1.262] *n* = 59	0.806 [0.753, 1.005] *n* = 38	0.904 [0.825, 1.020] *n* = 59
Interval coefficient of variation *CV_I_*	0.239 [0.186, 0.372] *n* = 55	0.332 [0.260, 0.394] *n* = 59	0.255 [0.183, 0.370] *n* = 38	0.307 [0.208, 0.376] *n* = 59
Harmonic spacing Δ*f_h_* (Hz)	117.0 [114.3, 117.4] *n* = 20	115.1 [114.0, 116.0] *n* = 20	119.1 [116.7, 120.1] *n* = 10	114.8 [112.4, 115.7] *n* = 20
Maximum sustained frequency *f*_max_ (kHz)	3.02 [2.92, 3.08] *n* = 14	4.20 [3.48, 4.64] *n* = 15	4.03 [—] *n* = 1	2.97 [2.88, 3.20] *n* = 18

Values are medians [first, third quartiles], with the parameter-specific sample size *n* shown in each cell. *n* denotes calls for individual-call parameters and observed sequences for sequence parameters. Eligibility differed among parameters. A dash indicates that an interquartile range is unavailable.

## Data Availability

The processed supporting data, statistical analysis scripts and documentation are included in Dataset S1. The complete original acoustic recordings comprise approximately 1.3 TB and are unavailable for online download because of their volume and current transfer limitations. Researchers may contact the corresponding authors, specifying the intended use, to arrange an on-site visit to Anqing Normal University to obtain the recordings.

## Supplementary Materials

The following supporting information can be downloaded at: https://www.mdpi.com/article/10.3390/ani16213340/s1. Table S1: Fish taxa and capture sources from the regional November 2023 survey in the Cambodian Mekong River; File S1: Cross-site acoustic alignment and shared-reception check; Dataset S1: Processed acoustic data, analysis code, and supporting documentation.
