# Supplementary material for: Shared Call Structure and Fine-Scale Spatial Variation in Calls Attributed to Boesemania microlepis in the Cambodian Mekong River
Source: Animals (Basel). 2026 Oct 23;16(21):3340. doi: 10.3390/ani16213340 (PMC13762187; doi:10.3390/ani16213340)
Supplement: Supplementary file 1 [file animals-16-03340-s001.zip › File_S1.pdf]

File S1. Cross-site acoustic alignment and shared-reception check

Supporting analysis for the revised manuscript animals-4611083 11 October 2026

This report documents an additional acoustic alignment and cross-reception check across the four Kampi recorders. East and Dock showed excess temporal coincidence and greater raw-audio similarity than unrelated-call controls, supporting partial shared reception. The estimates are conditional on the alignment and matching criteria. They do not constitute a complete shared-call census or an independently verified clock calibration.

Recording scope and evaluation design

The analysis covers the recorded portions of 9–19 November 2023, the common analytical period of the manuscript, and includes all six site pairs. It uses the final manually confirmed event table, including recovered missed calls, and original WAV header information. Unrecorded duty-cycle gaps are not treated as observations. The first recorder activation in each half-hour was used to define overlapping recording opportunities. Dock activations outside the other recorders' coverage were excluded. Filename times were converted consistently to Cambodian local time; this time-zone conversion is not a clock correction.

Half-hour blocks were assigned before fitting using a fixed SHA256 rule (Kampi-cross-site-20261011 followed by the block index; first digest byte below 128 for alignment estimation, otherwise evaluation). Assignment did not depend on event count or acoustic similarity. The alignment-estimation subset used 60 s margins at each common recording edge. Evaluation used the aligned overlap with 10 s margins at each edge. The East–Dock evaluation subset contained 259 blocks across all 11 dates, totaling 19.1347 h and 3,073 East events and 4,474 Dock events.

Acoustic alignment and timing limitations

Event-time differences were accumulated in daily lag histograms over ±50 s using 0.01 s bins and smoothing over 0.11 s. A linear lag trend was fitted only in the alignment-estimation blocks. The search allowed a midpoint intercept within ±35 s and a slope within ±3.5 s/day. For East–Dock, the fitted apparent lag, defined as Dock time minus East time, was 2.12 s at the midpoint near 14 November noon, with a slope of approximately +0.08 s/day. Predicted date-midpoint lags range from 1.72 s on 9 November to 2.52 s on 19 November. The grid resolution is not a demonstrated timing accuracy.

Acoustic lag includes both relative recorder timing and propagation delay. Numerical offset and drift records were unavailable for reconstructing an independently verified correction throughout deployment. A separate calibration-only search over ±0.60 s around the fitted trend attempted daily refinement using raw-audio comparisons. No date met the specified support requirement of at least four distinct calls in a ±0.06 s modal neighbourhood from at least three recording blocks. No daily refinement was therefore applied. The other five pairs did not yield similarly clear independent evaluation evidence, and their fitted search optima are not reported as device clock drifts.

Temporal coincidence and controls

Within each evaluation core, sorted event onsets were matched one to one after alignment, with a primary tolerance of ±0.20 s; no event was reused. Ten circular shifts of Dock event times within the same core, at −41.2, −31.1, −21.9, −13.3, −7.7, +7.7, +13.3, +21.9, +31.1 and +41.2 s, provided an accidental-coincidence benchmark while preserving counts and most local sequence structure. Excess coincidence was calculated as the actual match count minus the mean shifted-control count, divided separately by each recorder's evaluation event count. Uncertainty was summarized by 10,000 resamples of the 11 whole dates, with replacement, using seed 20261011. These intervals reflect between-date variability and do not incorporate alignment or same-source classification error.

Table S2. Primary East–Dock excess temporal coincidence estimates

Recorder	Evaluation events	Excess fraction	Date bootstrap 95% interval
East	3,073	5.65%	0.54–12.25%
Dock	4,474	3.88%	0.36–8.46%

There were 642 actual candidate matches and a mean of 468.5 shifted-control matches. Their difference, 173.5, is an expected excess, not an observed integer count of verified shared calls. The two recorder-specific fractions cannot be added to estimate a unique-source count or extrapolated directly to intervals when the recorders were not simultaneously recording.

Table S3. Temporal diagnostics for all six site pairs at ±0.20 s

Pair A–B	Actual pairs	Shifted mean	Excess A	Excess B
West–Middle	3303	3277.4	0.15%	0.17%
West–East	1307	1282.9	0.14%	0.35%
West–Dock	684	672.0	0.17%	0.29%
Middle–East	1382	1407.8	-0.18%	-0.38%
Middle–Dock	778	767.7	0.15%	0.22%
East–Dock	642	468.5	5.65%	3.88%

For all five pairs other than East–Dock, the date-bootstrap excess-coincidence intervals included zero. Negative excess values are retained as diagnostic fluctuations around the control benchmark, not interpreted as negative sharing. The absence of clear evidence does not establish zero shared reception, especially where a reliable relative timing correction could not be verified.

Table S4. East–Dock sensitivity to onset-matching tolerance

Tolerance	Excess fraction of East events	Excess fraction of Dock events
±0.05 s	0.07%	0.05%
±0.10 s	3.37%	2.32%
±0.20 s	5.65%	3.88%
±0.40 s	7.19%	4.94%

Raw audio corroboration and its limits

All 642 East–Dock evaluation candidates were processed from the original WAV recordings. For each of the other five pairs, 80 candidates were selected randomly using seed 2026101103. Each selected East or other A-site event was paired with one B-site event from a different date, preferring a similar local hour and duration, to provide an unrelated-call control. These are computational acoustic comparisons, not

independently labelled same-source examples. One East–Dock control excerpt contained insufficient audio; 641 controls and all 642 actual candidates were usable.

PCM fragments were resampled from 288 kHz to 12 kHz and filtered over 180–4800 Hz. Waveforms were compared within a 140 ms fine-alignment search around the separately detected boundaries. The composite score multiplied absolute normalized waveform correlation, positive correlation of 160 Hz-smoothed Hilbert envelopes at the waveform alignment, and square-root PSD cosine similarity over 180–3200 Hz. Thresholds were defined from the pooled unrelated-call controls across all six pairs, not optimized against actual candidate counts.

Table S5. East–Dock raw-audio diagnostics at two control thresholds

Pooled control quantile	Score threshold	Candidate passes	Control passes
95th percentile	0.080236	143/642	22/641
99th percentile	0.141479	24/642	1/641

The corresponding raw-compatible fractions of all evaluation events were 4.65% and 3.20% at East and Dock under the 95th-percentile threshold, and 0.78% and 0.54% under the 99th-percentile threshold. These fractions depend on the acoustic criterion. Stricter thresholds may miss weak or differently propagated observations of the same call, and the sensitivity of this same-source classifier has not been independently measured. Different-date controls may also differ from contemporaneous interference. Consequently neither criterion establishes an unbiased total shared-call fraction.

An additional diagnostic correction assumes that the unrelated-control pass probability represents acoustic false positives among accidental temporal coincidences. Under this unverified assumption, the 95th-percentile result becomes approximately 4.13% at East and 2.84% at Dock; the 99th-percentile result becomes 0.76% and 0.52%. These conditional diagnostics are retained in the accompanying tables for transparency and are not used as the primary estimate of true sharing.

Consequences for the manuscript

This additional check does not overturn the main descriptive findings presented in the submitted manuscript and retained in the revision, namely shared pulsed and harmonic call structure, late-afternoon and early-evening calling concentration, and spatial differences in received spectra and within-sequence repetition. The study's principal contribution remains the quantitative description of these structural, temporal and spatial patterns within a natural river reach. The confirmed event tables and the current revised acoustic-parameter estimates and statistical results are unchanged by this check. Its main consequence is to qualify source-level interpretations of the site contrasts; it does not establish that the observed differences reflect independent populations or exclusively differences in source behaviour.

The manuscript method and limitation descriptions are updated briefly in Sections 2.4 and 4.4. Shared reception and source distance remain interpretive limitations. Date-paired summaries do not demonstrate independent acoustic sources, and no cross-site candidates have been automatically deleted from the final event table.

Supporting records

The accompanying dataset includes the final block assignments, event-pair candidate ledger, per-block temporal controls, all six pair summaries, raw-audio comparison scores, both threshold choices, calibration-only daily refinement diagnostics, archived analysis scripts and a portable summary recalculation script. PCM fragment SHA256 values identify the original excerpts. The full raw recording archive is required to rerun acoustic comparisons and is available under the manuscript's Data Availability Statement; it is not duplicated in this supporting dataset.

Diagnostic figure

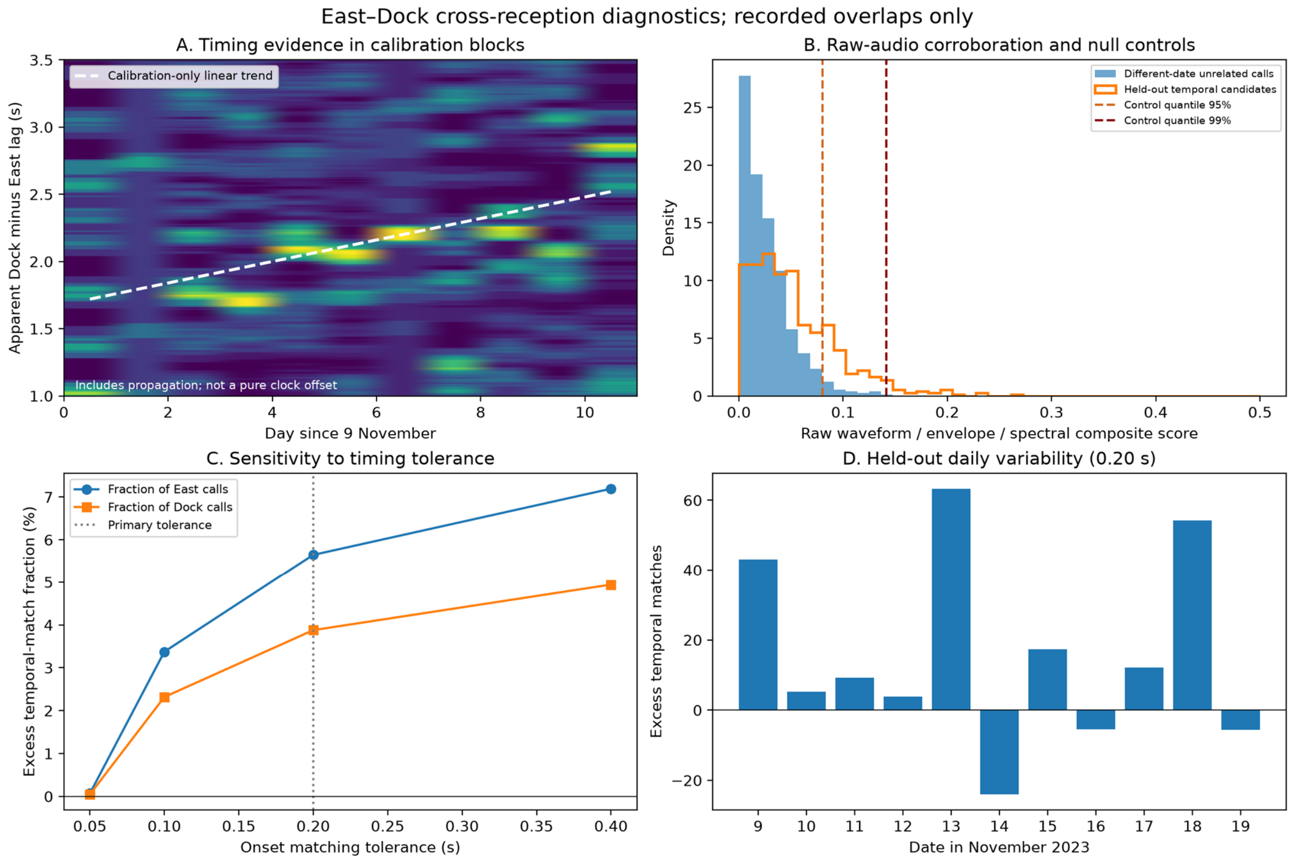

Figure S1. East–Dock diagnostic results. (A) Calibration-subset lag distributions and the fitted linear acoustic alignment, which includes propagation. (B) Raw-audio score distributions for held-out temporal candidates and different-date controls, with pooled-control 95th- and 99th-percentile thresholds. (C) Excess temporal coincidence fractions at four onset tolerances. (D) Date-specific excess matches at ±0.20 s, showing variability across the 11 dates.

The plotted score separation supports partial shared reception, while the tolerance sensitivity and date variation prevent treating one percentage as a precise, complete shared-call census.
