# Supplementary material for: Shared Call Structure and Fine-Scale Spatial Variation in Calls Attributed to Boesemania microlepis in the Cambodian Mekong River
Source: Animals (Basel). 2026 Oct 23;16(21):3340. doi: 10.3390/ani16213340 (PMC13762187; doi:10.3390/ani16213340)
Supplement: Supplementary file 1 [file animals-16-03340-s001.zip › Table_S1.pdf]

Table S1

Fish taxa and capture sources from the regional November 2023 survey in the Cambodian Mekong River.

No.	Taxon or field label	Research-team fish traps	Local fishers' net catches
1	*Acantopsis* sp.	Yes	Yes
2	*Acantopsis* sp. 1	No	Yes
3	*Acantopsis* sp. 2	Yes	Yes
4	*Amblyrhynchichthys micracanthus*	Yes	Yes
5	*Amblyrhynchichthys* sp.	Yes	No
6	*Barbonymus altus*	Yes	Yes
7	*Barbonymus gonionotus*	Yes	No
8	*Barbonymus schwanenfeldii*	Yes	Yes
9	*Boesemania microlepis*	Yes	Yes
10	*Brachirus harmandi*	No	Yes
11	*Cyclocheilos furcatus*	Yes	No
12	Cyprinidae-F06	Yes	Yes
13	Cyprinidae-F07	Yes	Yes
14	Cyprinidae-F08	Yes	Yes
15	Cyprinidae-F09	Yes	No
16	Cyprinidae-F10	Yes	Yes
17	Eleotridae indet.	Yes	No
18	*Hembarbus* sp.†	No	Yes
19	*Hemibagrus spilopterus*	Yes	Yes
20	*Hemibagrus wyckii*	Yes	Yes
21	*Henicorhynchus siamensis*	Yes	No
22	*Macrognathus facus*†	Yes	No
23	*Macrognathus siamensis*	Yes	No
24	*Mystus bocourti*	Yes	Yes
25	*Mystus singaringan*	No	Yes
26	*Pangasius elongatus*	Yes	No
27	*Pao cochinchinensis*	Yes	Yes
28	*Paralaubuca* sp.	Yes	Yes
29	*Parambassis wolffii*	No	Yes
30	*Pseudobagrus* sp.	Yes	Yes
31	*Pseudolais pleurotaenia*	Yes	Yes
32	*Puntioplites waandersi*	Yes	Yes
33	*Rasbora paviana*	Yes	Yes
34	*Rasbora tornieri*	Yes	No
35	*Thynnichthys thynnoides*	Yes	No
36	Unidentified goby	Yes	No

Yes and No indicate whether a taxon was recorded from each capture source during the survey. Taxa recorded from both sources are listed once.

Records are pooled across the regional survey conducted near the four acoustic monitoring sites. Genus- and family-level identifications and coded morphotypes follow the field records.

† Original field label retained.

Research-team traps yielded seven *Boesemania microlepis*: West 2, Middle 2, East 1 and Dock 2.
